# Point-of-Care Testing for Chlamydia and Gonorrhoea: Implications for Clinical Practice

**DOI:** 10.1371/journal.pone.0100518

**Published:** 2014-06-23

**Authors:** Lisa Natoli, Lisa Maher, Mark Shephard, Belinda Hengel, Annie Tangey, Steven G. Badman, James Ward, Rebecca J. Guy, on behalf of the TTANGO Investigators

**Affiliations:** 1 The Kirby Institute, University of New South Wales, Sydney, New South Wales, Australia; 2 The Burnet Institute, Melbourne, Victoria, Australia; 3 Apunipima Cape York Health Council, Cairns, Queensland, Australia; 4 Ngaanyatjarra Health Service, Alice Springs, Northern Territory, Australia; 5 Flinders University International Centre for Point of-Care Testing, Flinders University, Adelaide, South Australia, Australia; 6 Baker IDI, Alice Springs, Northern Territory, Australia; University of California Merced, United States of America

## Abstract

**Objectives:**

Point-of-care (POC) testing for chlamydia (CT) and gonorrhoea (NG) offers a new approach to the diagnosis and management of these sexually transmitted infections (STIs) in remote Australian communities and other similar settings. Diagnosis of STIs in remote communities is typically symptom driven, and for those who are asymptomatic, treatment is generally delayed until specimens can be transported to the reference laboratory, results returned and the patient recalled. The objective of this study was to explore the clinical implications of using CT/NG POC tests in routine clinical care in remote settings.

**Methods:**

In-depth qualitative interviews were conducted with a purposively selected group of 18 key informants with a range of sexual health and laboratory expertise.

**Results:**

Participants highlighted the potential impact POC testing would have on different stages of the current STI management pathway in remote Aboriginal communities and how the pathway would change. They identified implications for offering a POC test, specimen collection, conducting the POC test, syndromic management of STIs, pelvic inflammatory disease diagnosis and management, interpretation and delivery of POC results, provision of treatment, contact tracing, management of client flow and wait time, and re-testing at 3 months after infection.

**Conclusions:**

The introduction of POC testing to improve STI service delivery requires careful consideration of both its advantages and limitations. The findings of this study will inform protocols for the implementation of CT/NG POC testing, and also STI testing and management guidelines.

## Introduction

*Chlamydia trachomatis* (CT) and *Neisseria gonorrhoeae* (NG) cause sexually transmissible infections (STIs) that are fully curable with single dose treatment but are often asymptomatic for long periods of time [1]. Both infections can lead to serious complications [1] including pelvic inflammatory disease (PID) [2], ectopic pregnancy and tubal factor infertility [3], [4], and a range of adverse pregnancy and neonatal outcomes.[5], [6]

To interrupt infection transmission in populations at higher risk of CT and NG infection, and reduce the risk of sequelae, it is important to diagnose and provide treatment as early as possible in people with these infections [7]. A number of studies have found that with just a few weeks delay between testing and treatment, 2–3% of patients with CT infection have already developed PID [8], [9]. Timely diagnosis also enables contacts to be identified and treated through partner notification strategies.

In many clinical settings in remote areas of the world, distance from laboratories means there are significant delays in accessing diagnostics [10], [11], and in many resource poor countries, laboratory infrastructure is either not available or limited [12]. In the absence of diagnostic tests for STIs, the World Health Organisation recommends ‘syndromic management’ as the approach to diagnosing and treating common STIs [13]. This approach involves immediate treatment for STIs based on an algorithm of common signs and symptoms [14]–[16]. In remote parts of Australia, clinical services are located many hundreds, even thousands of kilometres, away from the laboratory, specimen transport may only occur once a week [17] and thus results may not be received from laboratories for 7–10 days. Typically, remote clinical services undertake syndromic management plus send specimens to laboratories for testing, with asymptomatic patients treated once the results are received from the laboratory.

However, syndromic management leaves many infections untreated as well as causing overtreatment in others [18], [19]. Over 80% of CT infections and 80% of NG infections in females and 50% in males are asymptomatic [20]–[22]. Thus only a small proportion of people with CT or NG present to clinical services with the symptoms that are required to [23] qualify for immediate treatment using the syndromic approach [14]–[16]. Furthermore, symptom-driven diagnosis has poor sensitivity and specificity for detecting infections, particularly in women. It is also difficult to develop algorithms with good sensitivity and specificity that are applicable in a range of primary care settings and across geographic locations [19], [24].

Point-of-care (POC) testing has the potential to improve diagnosis and management of STIs in areas with no or limited access to pathology services or in remote areas where there are considerable delays in receiving laboratory results. To date, use of CT and NG POC tests has been limited due to deficiencies of the available technology. For example, the only devices which have been commercially available have involved lateral flow and immuno-chromatographic detection and have exhibited poor sensitivity and specificity, most detect single infections only, and many are complicated to use [25], [26]. Recently, a molecular-based POC test system for the dual detection of CT and NG was developed [12]. The Xpert CT/NG assay (Cepheid) has very high sensitivity and specificity for these two tests, is easy to use, and results are available in 90 minutes. The CT/NG test was approved by regulatory bodies in the US, Europe and Australia in 2013. Other molecular based POC tests are under development [12].

This new generation of molecular POC tests provide an opportunity to expand the use of POC testing. However to our knowledge, no studies have examined the clinical implications of introducing CT/NG POC tests. In the context of the ‘Test Treat ANd GO’ (TTANGO) Trial [27], a randomised controlled trial of the GeneXpert in remote communities of Australia, we used qualitative methods to explore for the first time, the clinical implications of using CT/NG POC tests in routine clinical care.

## Methods and Materials

### Ethics statement

Ethical approval for the study was received from the West Australian Aboriginal Health Information and Ethics Committee, the West Australian Community Health Board Research Ethics Committee, the Townsville and Cairns Health Service District Human Research Ethics Committees and, the Aboriginal Health Research Ethics Committee of South Australia.

### Setting

Australia has extensive and high quality laboratory infrastructure, like other resource-rich settings, with numerous laboratories all using highly accurate PCR tests. STI testing is available at primary care centres, sexual health clinics and family planning clinics and other settings, with primary care diagnosing most STIs in Australia. STI testing is recommended for: (i) patients with symptoms or considered high risk, (ii) annually in young people and men who have sex with men (MSM), and (iii) 3–6 monthly in higher risk MSM. A repeat test at 3 months is also recommended to detect re-infections in any person diagnosed with an STI [28]–[30]. POC testing by PCR for STIs is not yet established in Australia.

In urban areas clinical services are staffed by doctors and nurses, and clinics can send a specimen to a laboratory and generally receive a result within a few days, with treatment occurring soon after. Specialist sexual health services also have access to POC microscopy for symptomatic patients.

In remote Australian Aboriginal communities, there are only generalist primary heath care centres, which are staffed by nurses and Aboriginal Health Practitioners mainly, with most having ‘fly in’ and ‘fly out’ Medical Officer support. Client mobility is typically high, and follow-up systems are not always effective, with 11–25% of people not treated for STIs in some areas, and the average time to treatment estimated to be 21 days for asymptomatic patients [31]. In remote Australian Aboriginal communities the prevalence of CT among 16–34 year olds was estimated to be 9% for CT and 7% for NG in 2010, and was highest in 16–19 year olds. This compares with 3–4% for CT in non-Indigenous youth aged 16–29 years and <1% for NG, making timely treatment even more important [32]–[34].

**Figure 1:**
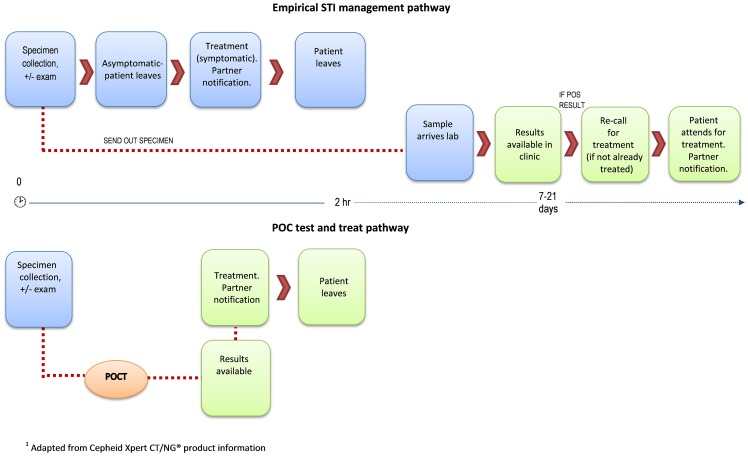
Indicative STI management pathway (remote) with and without point-of-care testing.

The current STI management pathway common in remote primary care health services is reflected in the top half of Figure 1. Standard processes for ordering STI tests and dispensing treatment vary according to jurisdiction, the professional qualification of health care workers and health service guidelines. For example, in some very remote services, Registered Nurses are able to administer STI treatments presumptively or on basis of a test result according to standard protocols, and without a doctor's order. Also Nurse Practitioners may be accredited to assess patients for STIs, order pertinent tests and provide presumptive treatment according to clinical practice guidelines. In addition, standing orders allowing antibiotic treatment are often in place to compensate for the absence of medical staff where necessary.

### Study design

Between March and August 2013, in-depth qualitative interviews were conducted with key experts to explore, among other issues, the likely impact of CT/NG POC testing on current clinical practice. The focus was on POC testing generally and not limited to molecular type tests, although molecular tests were often the focus of discussion.

### Participants

Purposive sampling was used to identify participants with relevant sexual health and/or laboratory expertise. We sought to interview male and female participants with remote and urban expertise, broadly representative of the eight Australian states and territories.

### Interviews

Interviews were conducted by LN via telephone, skype or, where possible, in person, and took between 30 and 75 minutes. Written informed consent was obtained from all participants.

### Data management and analysis

Recruitment continued until the data were saturated or no new themes emerged [35]. Interviews were digitally-recorded, transcribed verbatim and transcripts were later checked for accuracy against the recordings and to ensure familiarisation prior to analysis. Transcripts were uploaded into QSR Nvivo (Version 10), a qualitative data management and analysis program (QRS International PTY Ltd, Melbourne, Australia). Each transcript was systematically coded and content analysis was performed to examine frequencies of recurring codes and to allocate salient themes [36].

## Results

Participants (n = 18) had sexual health and/or laboratory expertise and included sexual health physicians and nurses in urban, regional and remote health services (n = 8), academics (n = 2), policy makers (n = 4), and laboratory based microbiologists (n = 4). The majority of participants were male (56%) with the average age being 49 years (range 39–58 years). Participants were drawn from five of the eight Australian States and Territories. Nine of the participants were urban based, six worked across both urban and remote settings and three worked predominantly in remote communities.

Participants highlighted the potential impact POC testing would have on different stages of the current STI management pathway in remote Aboriginal communities (upper half of Figure 1) and how the pathway would change (lower half of Figure 1). The results are summarised according to different thematic components of the STI management pathway.

### Offering a POC test

Several participants highlighted there may be some novelty attached to POC testing and staff may need to be prepared in order to manage expectations for testing from patients who may be curious about POC testing but not necessarily at risk for STIs.

[You could have] *anyone coming in and going “I want that test” when actually they don't fit, they're not at risk and it's just a curiosity and the clinic staff have to manage that and they have to know how to manage that (Participant #15).*

Some suggested that staff may have a lower threshold for testing, or be more inclined to offer testing without justification, purely because of the convenience of having the test on site.

Others expressed particular concerns about testing in children/adolescents.

*[S]ometimes they [staff new to remote settings] do tests when it's inappropriate … I just think there has been concern about doing … STI screens in kids, looking at things - well if it's negative they haven't been sexually abused that type of thing, so you just need to make sure that that tendency doesn't increase (Participant #13).*

Participants noted the importance of appropriately targeted testing to ensure efficient use of scarce resources. This consideration is already pertinent under current practice, as one participant noted.

*[A]dult health checks* [an Australian government initiative that targets Aboriginal people (15–54 yrs) to facilitate early detection, diagnosis and management of common, treatable conditions] *have been a huge facilitator to increasing the uptake of STI testing in general but … when you look at a lot of those remote communities more than a third of the testing is being done in people over the age of 40* (Participant #15).

Participants highlighted the need for clear protocols to guide appropriate POC testing of both index cases and contacts.

*So we have to make sure that people are trained properly, not just about the point-of-care test, but about the whole thing and when it's appropriate to do an STI test and when it's not (Participant #13).*

Some participants felt that practitioners would also need to understand POC test characteristics and limitations (given that sensitivity and specificity may differ from routine laboratory tests), depending on the purpose of testing.

*I find that practitioners in general have very low levels of knowledge around screening, but when you actually talk to them … they're all screening for something and they're not thinking about that as a screening test and they're not thinking about the difference between what you test and what information goes with it and how much time you spend doing it in a context of screening, as opposed to in a context of doing a diagnostic test (Participant #15).*

Some participants felt that for the full benefits of POC testing to be attained (such as extending the reach of testing via non-medical staff in remote services), consideration may need to be given to which health service staff have authority to initiate testing. Potentially there are implications for clinical guidelines and legislative change, as in some Australian jurisdictions pathology request forms can only be signed by a medical practitioner. In the context of POC testing, it was felt that the process for authorisation of testing needs to be more ‘rapid’.

*[A]ll health workers that I've come across are in principle able to … collect the specimen; it's the doctors that request the tests, so again you might need to have some sort of review of who is requesting the test. I don't think it needs to be a doctor … in some places … doctors have refused to sign pathology forms when the nurses and health workers are perfectly capable of screening people … I think that probably ties into the legislation for the health workers and nurses about what they are able to do (Participant #15).*

Some participants suggested that the availability of POC testing might help to raise the profile of STIs and increase testing rates in health services where sexual health is not a high priority.

*So in a place where … screening isn't happening consistently then I think it would be … great value … particularly in remote settings … I think a test like that would actually increase their [nurses] confidence around some management issues … increase their interest if you like (Participant # 15).*

### Specimen collection

Depending on current practice within the service, participants noted that consideration would need to be given to the timing of specimen collection, and whether this happens before or after the clinical consultation. A number of participants felt that specimen collection could be integrated into processes where urine would usually be collected.

*[I]t could be simply “oh you're here for a health check-up, before we start the health check-up can we have a urine sample?” and then while you're doing the health check the test is being processed (Participant #4).*

*I think people would be okay with that. They go and pee, and we can get the test going while we're doing some talking, … we also know that most people don't want to do much talking about sex, so you know, “I just want to know if I've got something”. So I think from a patient perspective, I think it's very patient-oriented, this approach (Participant # 9).*

The process by which patients return specimens to clinicians may also need consideration, although this may not necessarily differ greatly from standard practice.

### Conducting the POC test

Several participants questioned the logistics of locating test equipment and consumables within smaller remote services, where bench and storage space may be limited. Depending on the test device, there may be need for a continuous power supply, refrigeration and internet connectivity. Maintaining confidentiality of patient results may also influence location of the test device.

*[F]or the HIV tests we're doing [for MSM in urban settings], they're just a strip, but you need an area where you can sit that strip for it to be incubating where the patient's not sitting on top of it, waiting for the result. So you would need to think about how big the machine is and where it's going to go and is it okay that it sits in the room with you while you do the rest of the things and then [it] goes, ‘beep’? (Participant #10)*

### Syndromic management for STIs

Some participants suggested that symptomatic patients should continue to be treated in accordance with syndromic management protocols.

*[I]f someone is symptomatic should they or should they not have a POC test? Should they just have an ordinary PCR and also have a broader battery of tests. But particularly for remote Aboriginal communities in WA [Western Australia], not to approach that person from a syndromic paradigm would probably be unethical really (Participant #3).*

Alternatively, others suggested that syndromic management may become less applicable, as POC testing would help guide management and result in more targeted prescribing.

*[I]t does allow you to move from a syndromic approach, which is basically treating someone just because they're a contact or because they've got symptoms, to treating them based on whether they have been diagnosed formally … or not (Participant #17).*

There was an understanding among participants that current treatment algorithms may need to alter slightly. For example, in relation to management of vaginal discharge, a negative chlamydia or NG result would need to direct clinicians to treat for other likely infections such as candida, trichomoniasis and bacterial vaginosis.

*Look, it'll probably change those algorithms a bit, what will go in there is test for these things, if these things are negative then do this, I suppose. Which is treatment for BV [bacterial vaginosis] and trich [trichomoniasis] and anything else that may cause it (Participant #10).*

Treatment algorithms for urethral discharge in men may also need to change. However, as diagnosis and treatment of urethritis in men is generally well managed, one participant wondered whether this might be undermined by POC testing. For example urethritis can be caused by *Mycoplasma genitalium* which is not tested for but often treated inadvertently due to the antibiotics given syndromically for CT/NG.

*[I]f anything I think your risk in men with urethritis is under treatment, because at the moment they're treating everyone pretty much on spec and before they get a test result, so … if people were going to start using it [a POC test] and then use it as a reason not to treat, that could be a potential risk for men (Participant #15).*

In some jurisdictions, treatment guidelines recommend dual therapy (for both CT and NG) in cases of urethral discharge. However one participant queried whether treatment for both CT and NG should be given in the case of a CT only positive POC result. Some participants noted that the local epidemiology would be an important consideration in this instance.

*[I]f someone in a remote location had urethral discharge- I'm not familiar with the current version of the CARPA manual [guidelines for clinical practice used in many remote Australian communities] for example, but I expect they're offering treatment for gonorrhoea and chlamydia together … [so] if you do a point-of-care test and it shows they've got chlamydia, not gonorrhoea, would you still treat them for gonorrhoea? So would that change that? (Participant # 9)*

### PID diagnosis and management

There was a suggestion that PID is generally underdiagnosed in remote settings and that POC testing might heighten awareness of health professionals and strengthen management of these cases.

*I think it would reinforce management [referring to PID] in people's head as well, when you've got something immediate, you know, if it's a week down the track then people… might forget the details of that case, but if you've got it there immediately and you thought “oh yeah okay that's what it is” (Participant # 15).*

At the same time, the potential to rely too heavily on a POC test was highlighted by some participants, particularly in relation to the differential diagnosis of pelvic pain.

*[Y]ou're not just talking about chlamydia and gonorrhoea, you are actually talking about what the test means in the context of who and what presenting symptoms they've had … it's not just here's your test and it's about the test, it's actually about your predictive value of using that test depending on who you are testing … and getting across that understanding of how you interpret it … related to the individual that they're testing basically or the age group if you like (Participant #15).*

Some participants also questioned whether PID treatment algorithms would change in the context of a POC test.

*But in terms of, for example, the PID treatment, if you did a swab on a woman with pelvic pain and did a point-of-care test that showed they had Chlamydia, I think that you'd be treating for PID, which covers Gonorrhoea treatment (Participant # 9).*

### Interpretation and delivery of POC results

Participants identified counselling and provision of test results to patients as a key area of practice that would be potentially impacted by POC testing, with the process being condensed into a much shorter timeframe than usual.

*[P]eople may feel … a bit challenged by that, and that thought of not being able to … process that information or have time to think about it may actually be a bit of a concern. It … certainly adds a layer of complexity for the health care provider to explain … what the ins and outs of the test are, what the possibility of the false negatives, the false positives, what the treatment options are (Participant #7).*

Participants noted that providing results on the same day as testing could result in the capacity to free up appointments and reduce the resourcing and effort that goes into client follow up.

*[F]or a service such as ours, obviously … you're freeing up appointments … for other people if we can manage in a single consultation that diagnosis and management (Participant # 7).*

*[W]ell, communicating results to patients- … If you can do it on the same day … there's less work to be done in either ringing people, chasing people, bringing people back for review, that kind of thing. It wouldn't eliminate it, but it would reduce that work quite substantially (Participant # 6).*

During routine STI testing it is accepted best practice that clinicians should conduct a full STI screen involving HIV and syphilis in the context of a positive CT or NG result. One participant identified that POC testing may provide an opportunity for increased patient engagement and that this might open the door for a more comprehensive approach.

*[W]hen you do their sexual history potentially they are under-reporting self-report stuff and then when there is a positive result it does provide an opportunity to have a more frank discussion about what else is going on in people's lives. And then the opportunity to test for other … STIs like HIV that you otherwise would not have done (Participant #11).*

### Provision of treatment

While immediacy of treatment was recognised by many to be a fundamental benefit of POC testing, some participants felt that a review of policy or legislation may be necessary in some jurisdictions in order to facilitate timely dispensing of medication by non-medical staff.

*[I] think we have standing orders for instance for nurses to be able to give Azithromycin in very defined circumstances so they can supply the medication that's … prescribed to the doctor. But I think we need to look nationally at how we can facilitate that supply of medication by healthcare professionals other than doctors. So – because otherwise … it defeats the point … (Participant # 7).*

In addition to informing clinicians about notifiable infections, it was noted that some laboratory service providers guide clinicians in treatment options, via comments provided on laboratory test results. In the absence of laboratory testing this guidance would no longer be available, and health professionals would need to have a heightened working knowledge of treatment guidelines.

*[T]he way we inform a lot of clients is through the comments … that we put on reports, for instance our positive gono PCR reports talk about what antibiotics you should use depending on what region of Western Australia you're actually in; so it actually complies with our Silver Book [a standard treatment manual based on best practice and the syndromic approach] of STI guidelines. They would miss out on all that, so there's an educational aspect that could be lost if they weren't still doing standardised laboratory testing at the same time (Participant #17).*

### Contact tracing

Participants identified contact tracing as a key area of practice that would be impacted by POC testing. Patients will need to be prepared for the possibility of discussing sexual contacts and possibly having them notified in a much shorter time frame.

*[N]ormally … if we're not using a point of care test, then we go, “Your test is positive, … here's what you've got to do, let's help you …” and sometimes that decision to tell the partner takes a long time. How is that going to work if you've got a positive test, you and me behind closed doors and your partner's sitting outside? And is there space for that [for people to chat together in private]? … There could be violence that comes from partners being told right there and then (Participant #10).*

As noted by the participant above, the potential for violence exists between sexual partners, when one person in a relationship is diagnosed with an STI. However participants also indicated that having a definitive test result - as opposed to treating on the basis of symptoms (which lacks the specificity of laboratory tests) - might avert unnecessary notification of partners (and the associated potential for violence or other social implications). In turn, this could free up staff time that would otherwise be dedicated to contact tracing. The possibility for partner delivered therapy was raised as an additional consideration.

### Management of client flow and wait time

The ease of performing the POC test, the turnaround time for the test result and how simply POC testing could be integrated into routine workflow were important considerations for many participants, though it was recognised that this would be largely influenced by the type of test. In particular, participants highlighted how clinical workflow can be impacted by a positive test.

An important consideration highlighted by one participant is how critical it is for the test result to be read at a specific time point.

*[H]ow important is it that its read at exactly the right time for instance … that may never happen because there are all other contingencies that might come up and drag people [staff] away from the test (Participant # 2).*

Participants recognised the opportunity for education and engagement (on a range of health issues) that arises through POC testing as patients wait for their test results.

*[T]hat opportunistic engagement around a whole range of health issues is potentially important. Anything that you can sell to the community that may make it more attractive to present at a service is going to be beneficial for a whole range of reasons (Participant # 11).*

In particular, taking the opportunity to raise awareness about STI prevention in the context of a negative test result was highlighted.

*[E]ven in remote areas and with high rates … then you will still have a lot of people who are getting a negative result, whereas normally we don't spend a lot of time on thinking about that. So what the doctor normally says with your HIV test is “oh that was fine” and straight on to the next thing, rather than – I mean that person could of gone through quite a bit of angst and been at quite a bit of risk, and equally so could your person who's been screened, you need to weigh on them, you didn't get it this time, but you could next time (Participant #5).*

### Re-testing at 3 months after infection

In the context of non-medical or less experienced staff (eg. fly-in nurses) having greater ability to initiate STI testing, many participants emphasised the need for clear protocols to provide guidance about appropriate re-testing timeframes.

*[W]hat you don't want is people retesting two or three days later and finding the PCR [point of care test] is still positive thinking that therefore they've had a failure of treatment when in fact all they've got is carry over DNA. So they have to know things like how soon should you repeat it, who should you repeat it on … [otherwise] you could tie yourself in knots chasing your own tail. You could be diagnosing relapses and reinfections that actually don't occur (Participant #17).*

## Discussion

Our study has identified that the introduction of POC testing to improve STI service delivery requires considerable forethought and planning [37], [38]. While stakeholders identified a range of potential benefits of using this technology, they also highlighted that the integration of CT/NG POC testing to remote clinical practice needs careful consideration. Potentially it will result in some changes to the STI management pathway, and policy and clinical guidelines may need to be altered in response.

Participants identified a range of benefits of using POC testing in remote settings including more timely and targeted prescribing, particularly in the context of increasing NG resistance [39], freeing up staff time usually spent on follow-up of cases who haven't returned for treatment, greater opportunities to offer a full STI screen for people with a positive test, more timely and targeted contact tracing, and more timely information to guide PID diagnosis and management. More targeted treatment is a common benefit raised in regards to POC testing, but other benefits were quite specific to CT and NG testing and management. The TTANGO trial aims to determine if these benefits will be realised.

A number of potential challenges were also raised. Many of these could be overcome by appropriate POC resources, training and support relating to the communication of results, logistics of testing and location of the POC device, and planned flow of patients through the clinic from the time of initial assessment through to treatment uptake (where indicated). However other issues raised will need careful discussion and planning. The potential to conduct ‘opportunistic’ POC testing, in populations that would not otherwise be tested raises the importance of ensuring there are systems in place to ensure testing is only conducted by trained operators in response to a formal test request and systems are in place to address how to manage a positive result. Also legislative changes may be needed if Aboriginal health practitioners are to conduct most STI testing and there is a preference for them to be able to request tests and provide treatment. As Aboriginal health practitioners are often the main care providers in remote services, the need for them to have greater authority in relation to initiating tests and providing treatment (according to guidelines) similarly applies to other areas of chronic disease and preventative health.

As the POC assay being used in TTANGO (Xpert CT/NG) has sensitivity and specificity equivalent to the routine laboratory tests [40], [41], treatment algorithms may need revision. For example, in the case of CT-only diagnoses, there would need to be clarity around treatment and whether, depending on local epidemiology, treatment for NG should also be provided (as per some current guidelines). This is an important consideration in the context of increasing antibiotic resistance by NG [39]. Also changes to syndromic management guidelines might be needed, but there would need to be careful consideration of whether syndromic management is completely stopped or altered. POC test performance characteristics and local disease epidemiology would be important considerations. For example, syndromic management of vaginal discharge is poorly predictive of CT or NG infection, and a highly sensitive and specific molecular POC test might facilitate more targeted treatment in this instance. If CT and NG are effectively excluded by a POC test, then metronidazole plus an anti-candida cream should cover the remaining likely causes; specifically bacterial vaginosis, trichomonas, and candida. However, over-riding syndromic management of urethral discharge in men may be less straightforward. As several participants in the study noted, other organisms (such as *M genitalium*) are implicated in non-gonococcal urethritis [42], so treatment of symptomatic men will probably still be required, though an anti-gonococcal drug may no longer be necessary. Similarly for contact tracing, where usually contacts are treated presumptively, guidelines may need to be reviewed.

Our study has several limitations. The qualitative approach, small sample size and the non-random nature of the sampling strategy limit the generalisability of our findings. However, to overcome this, we purposively sampled a broad range of recognised experts from different disciplines and jurisdictions with expertise in the testing and diagnosis of STIs. Participant responses were influenced by participant expertise and familiarity with specific work environments, and may not reflect the real-life/on-ground situation in all health services. If POC testing is introduced in the future, an assessment would need to be made in each health service setting, to determine the necessary changes to clinical practice and guidelines to incorporate POC testing into practice and health service guidelines and protocols. Although the interviews focussed on remote Australian Aboriginal communities, the findings have relevance for STI clinical practice generally.

In conclusion, the findings of this qualitative research will inform guidelines related to implementation of CT/NG POC testing, similar to those already available for HIV, syphilis and malaria POC testing [43] [44] but tailored to specific issues related to CT/NG identified here. These findings will also guide policy makers should they wish to plan for POC test implementation. Specifically, findings may inform: consideration of POC test logistics in remote services (such as test location, power supply, refrigeration, and internet connectivity); the need to review existing legislative processes to facilitate timely generation of pathology requests by non-medical staff; revision of syndromic management algorithms; and, training considerations relevant to the integration of STI POC testing in remote clinical practice.
